# Association between dietary intake of flavonoids and chronic low back pain: a cross-sectional study

**DOI:** 10.3389/fnut.2024.1436461

**Published:** 2024-10-03

**Authors:** Haibin Zhou, Yang Xi, Sizhe Gao, Yan Zhou

**Affiliations:** Department of Pain Management, Beijing Jishuitan Hospital, Capital Medical University, Beijing, China

**Keywords:** flavonoid, chronic low back pain, National Health and Nutrition Examination Survey, association, cross-sectional study

## Abstract

**Aim:**

The purpose of this study was to explore the association between flavonoids intake and chronic low back pain (CLBP).

**Methods:**

This cross-sectional study analyzed data from the National Health and Nutrition Examination Survey. Dietary flavonoids intake was assessed using a two-day recall questionnaire on dietary intake. CLBP was defined based of self-reported question. Weighted univariate and multivariate logistic regression models were performed to evaluate the relationship between flavonoids intake and CLBP. Additionally, subgroup analyses were conducted based on age, sedentary behavior time, arthritis, depression, and sleep disorder.

**Results:**

A total of 3,136 adults were included, and 460 participants developed CLBP. After adjusting confounders, compared with the lowest total flavonoids intake tertile (reference group), flavonoids intake with highest tertile (>170 mg) was associated with reduced odds of CLBP [odds ratio (OR) =0.74, 95% confidence interval (CI): 0.57–0.95]. This relationship of flavonoids intake with CLBP remained statistically significant among participants aged ≥45 years (OR = 0.52, 95%CI: 0.35–0.76), with sedentary behavior time of >3 h (OR = 0.60, 95%CI: 0.41–0.86), with arthritis (OR = 0.51, 95%CI: 0.29–0.90), depression (OR = 0.48, 95%CI: 0.24–0.98), and sleep disorder (OR = 0.27, 95%CI: 0.12–0.60).

**Conclusion:**

Higher flavonoids intake was found to be negatively associated with the likelihood of CLBP. For the general adult population, consuming foods rich in flavonoids may be linked to a reduced risk of CLBP.

## Introduction

Chronic low back pain (CLBP) is a common health issue, affects physical activity and lowers an individual’ quality of life (1). According to reports,70–80% individuals in the general population are likely to experience low back pain (LBP) at some point in their lives (2, 3). The costs associated with CLBP are substantial, imposing a significant financial burden on patients and their families. Therefore, identifying the factors linked to CLBP holds immense importance for its management and prevention. CLBP, as a painful condition related to the musculoskeletal system, was associated with multiple factors, such as body mass index (BMI), physical activity, depression (4), and socioeconomic levels (5). The cause of CLBP is complex, and it has been widely acknowledged that intervertebral disc degeneration plays a significant role in the development of CLBP (6).

Flavonoids, a class of natural phytochemicals present in plants, have long been recognized for their potent antioxidant properties (7). Previous studies have suggested that flavonoids may potentially ameliorate intervertebral disc degeneration and its associated pain, primarily attributed to their anti-inflammatory and antioxidant mechanisms (8–10). Flavonoids have been found to exert analgesic properties by means of their anti-inflammatory effects, activation of opioid receptors, suppression of prostaglandin synthesis, and various other mechanisms (11–13). In addition, the consumption of flavonoids has the potential to ameliorate risk factors associated with CLBP. For instance, Song et al., pointed out that flavonoids possess the ability to directly inhibit weight gain through biologically active metabolites (14). A systematic review and meta-analysis also demonstrated that flavonoids may improve symptoms of depression and anxiety, attributed to their anti-inflammatory and antioxidant properties (15). However, the relationship between flavonoids intake and CLBP has not been extensively studied until now.

Based on this background, we conducted a cross-sectional analysis to explore the association between flavonoids intake and CLBP utilizing data from the National Health and Nutrition Examination Survey (NHANES), with the objective of enhancing its management through dietary guidance.

## Methods

### Study population

This cross-sectional study involved participants from the NHANES 2009–2010. NHANES is an annual cross-sectional survey that is conducted nationally by the US National Center for Health Statistics (NCHS) (16) NHANES uses a complex multistage probability sampling to evaluate the health and nutritional status of the general US population, with a data collection process of interview and laboratory examinations. NHANES study received approval from ethics review committee of the NCHS, and all participants provided written informed consent.

**Figure 1:**
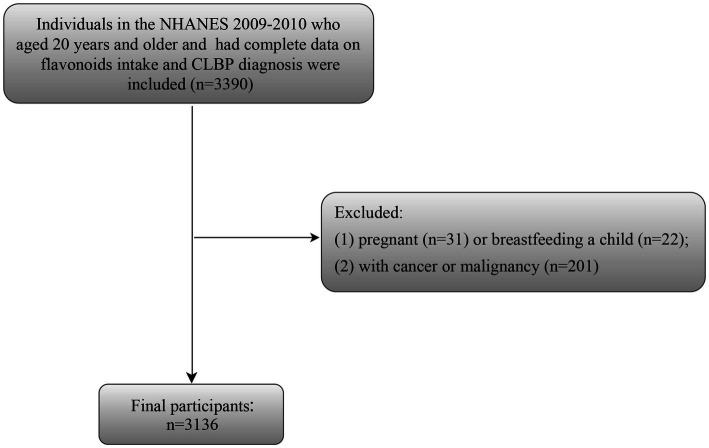
Flowchart of subjects’ selection.

Given that NHANES only releases the data on flavonoids dietary intake and CLBP for the cycle of 2009–2010, the selection criteria for the study population are based on this cycle data. Inclusion criteria: (1) individuals aged 20 years and older; (2) individuals had complete data on flavonoids intake; (3) individuals had complete data about CLBP diagnosis. Of these, we eliminated some participants who were pregnant (*n* = 31) or breastfeeding a child (*n* = 22), and had cancer or malignancy (*n* = 201). Finally, 3,136 participants were selected for further analysis (Figure 1).

### Data collection

#### Assessment of chronic low back pain

Participants was classified as CLBP when they had to answer “yes” to follow three questions (17): “Have you ever had pain, aching or stiffness in Back/Neck/Hip almost every day for at least 6 weeks in a row?” “Was there one time when you had pain, aching or stiffness in your low back on almost every day for 3 or more months in a row? and “Do you still have pain, aching or stiffness?”.^1^

#### Assessment of flavonoids intakes

Flavonoids intake was downloaded from Flavonoids Values for US Department of Agriculture Survey Foods and Beverages. In this study, the average dietary flavonoids intakes from two 24 h recalls were used as final dietary flavonoids intake. Flavonoids intakes were classified into three levels according to tertiles: ≤38 mg (the first tertile), >38 and ≤ 170 mg (the second tertile), and > 170 mg (the third tertile). The NHANES database contains detailed information on 29 specific flavonoids, which are categorized into six groups: Isoflavones, Anthocyanidins, Flavanones, Flavonols, Flavones, and Flavan-3-ols. Intake levels of each subtype of flavonoids were classified into three categories based on tertiles.

#### Possible of covariates

Information of participants was collected from NHANES database: age (years), gender, race, education, marital status, poverty income ratio (PIR), smoking status, drinking status, physical activity, sedentary behavior time (h), BMI, (kg/m^2^), obesity, arthritis, depression, sleep disorder, hypertension, diabetes, dyslipidemia, cardiovascular disease (CVD), chronic kidney disease (CKD), total energy (kcal), C-reactive protein (mg/dL), glucocorticoids, analgesics, and osteoporosis. Sleep disorder was assessed based on the following questions (18): “Have you ever told a doctor or other health professional that you have trouble sleeping?” Participants were classified as having diabetes if they had a self-reported physician diagnosis, or were taking medication for hypoglycemic drugs, or had a fasting glucose of ≥126 mg/dL, or had glycated hemoglobin (HbA1c) of ≥6.5% (19). Participants were classified as having dyslipidemia if they had total cholesterol (TC) level of ≥200 mg/dL, triglyceride (TG) level of ≥150 mg/dL, low-density lipoprotein cholesterol (LDL-C) level of ≥130 mg/dL, high-density lipoprotein cholesterol (HDL-C) level ≤ 40 mg/dL, or self-reported hypercholesterolemia, use of cholesterol-lowering therapy, or administration of lipid-lowering drugs. Arthritis was assessed based on the following questions (20): “Has a doctor or other health professional ever told you that you had arthritis?” Participants were asked to choose between two response options: “Yes” or “No.” To further distinguish between types of arthritis, individuals who responded positively were asked to specify the type, which included categorizations such as “Rheumatoid arthritis,” “Osteoarthritis or degenerative arthritis,” “Psoriatic arthritis,” and “Other.”

### Statistical analysis

The missing proportion of “Physical activity” and “Total spine bone mineral density” is relatively high, thus these missing variables were classified as “Unknown.” Other missing variables were imputed using the random forest chain equation multiple interpolation method. Supplementary Table S1 shows a sensitivity analysis on the data before and after interpolation. Considering the complex sampling design of NHANES, we employed the appropriate weights to the data in accordance with NHANES guidelines. WTDR2D was employed when conducting an analysis utilizing the smaller sample with completed Day 1 and Day 2 dietary data, as recommended by NHANES. In this study, 3,136 individuals were adjusted for accuracy to represent a population of 146,092,167. Continuous variables were presented as weighted mean standard error (SE), using independent-samples *T* test to compare differences between groups; categorical variables were expressed as frequencies and percentages, and compared using the Rao-Scott Chi-Squared test. All statistical analyses were conducted using SAS 9.4 software.

Weighted univariate and multivariate logistic regression models were then used to determine the relationship between total flavonoids and each subtype of flavonoids intake with CLBP. Model 1 was adjusted for no covariates; Model 2 was adjusted for age, gender, and race; Model 3 was adjusted for age, gender, race, PIR, arthritis, depression, sleep disorder, and analgesics (Supplementary Table S2). Odds ratio (OR) with 95% confidence interval (CI) was calculated. *p* < 0.05 was considered as significant. In addition, subgroup analyses were conducted based on age (<45 years/≥45 years), sedentary behavior time (≤3 h/>3 h), arthritis (No/Yes), depression (No/Yes), and sleep disorder (No/Yes).

## Results

### Characteristics of participants

Table 1 shows the characteristics of the study population. The mean (SE) age of the 3,136 participants was 42.27 (0.37) years, with 50.77% of male participants. 460 participants were classified as CLBP. We also compared the characteristics of participants between CLBP group and non-CLBP group. Compared to the non-CLBP group, participants with CLBP tended to be older, Non-Hispanic White, less likely to have attained a college degree, had lower PIR, and were more likely to be obesity. Besides, CLBP participants were more likely to be smokers and have comorbidities like arthritis, depression, sleep disorder, hypertension, diabetes, and CVD (*p* < 0.05). It was observed that CLBP participants may have lower proportion of dietary flavonoids intakes than those with non-CLBP.

**Table 1:** Characteristics of participants in the cohorts.

Variables	Total (*n* = 3,136)	Non-CLBP group (*n* = 2,676)	CLBP group (*n* = 460)	Statistics	*p*
Flavonoids intake, mg, Mean (S.E)	222.83 (18.53)	222.78 (18.15)	223.14 (44.95)	*t* = −0.01	0.993
Flavonoids intake, *n* (%)				χ^2^ = 7.58	0.023
≤38 mg	1,091 (33.25)	926 (32.70)	165 (36.74)		
38 ~ 170 mg	1,073 (33.43)	914 (33.09)	159 (35.52)		
>170 mg	972 (33.32)	836 (34.21)	136 (27.74)		
Age, years, Mean (S.E)	42.27 (0.37)	41.84 (0.40)	44.96 (0.82)	*t* = −3.67	0.002
Age, years, Mean (S.E)				χ^2^ = 6.93	0.008
<45	1,640 (55.72)	1,455 (57.28)	185 (45.92)		
≥45	1,496 (44.28)	1,221 (42.72)	275 (54.08)		
Gender, *n* (%)				χ^2^ = 0.09	0.769
Male	1,549 (50.77)	1,334 (50.89)	215 (49.97)		
Female	1,587 (49.23)	1,342 (49.11)	245 (50.03)		
Race, *n* (%)				χ^2^ = 8.28	0.016
Non-Hispanic White	1,344 (64.50)	1,105 (63.47)	239 (70.94)		
Non-Hispanic Black	603 (12.61)	521 (12.73)	82 (11.93)		
Others	1,189 (22.89)	1,050 (23.80)	139 (17.14)		
Education, *n* (%)				χ^2^ = 13.67	0.001
Below high school	839 (17.33)	708 (16.91)	131 (19.95)		
High school	703 (22.54)	585 (21.27)	118 (30.44)		
University graduate and above	1,594 (60.14)	1,383 (61.82)	211 (49.61)		
Marital status, *n* (%)				χ^2^ = 9.90	0.007
Married	1,613 (54.38)	1,406 (55.31)	207 (48.56)		
Never married	681 (23.33)	601 (23.69)	80 (21.07)		
Others	842 (22.29)	669 (21.00)	173 (30.37)		
PIR, *n* (%)				χ^2^ = 12.06	0.002
≤1.3	1,083 (23.39)	887 (21.95)	196 (32.44)		
1.3 ~ 3.5	1,143 (35.45)	981 (35.43)	162 (35.60)		
>3.5	910 (41.15)	808 (42.62)	102 (31.96)		
Smoking, yes, *n* (%)	1,359 (42.29)	1,081 (40.23)	278 (55.15)	χ^2^ = 11.87	<0.001
Drinking, yes, *n* (%)	2,364 (79.32)	1999 (79.10)	365 (80.70)	χ^2^ = 0.48	0.488
Physical activity, *n* (%)				χ^2^ = 8.00	0.018
<450 MET· min/week	292 (9.66)	245 (9.72)	47 (9.27)		
≥450 MET· min/week	2092 (70.71)	1826 (71.86)	266 (63.48)		
Unknown	752 (19.63)	605 (18.42)	147 (27.25)		
Sedentary behavior time, *n* (%)				χ^2^ = 2.36	0.124
≤3 h	1,132 (30.91)	980 (31.54)	152 (26.98)		
>3 h	2004 (69.09)	1,696 (68.46)	308 (73.02)		
BMI, kg/m^2^, Mean (S.E)	28.80 (0.19)	28.53 (0.22)	30.52 (0.46)	*t* = −3.91	0.001
Obesity, yes, *n* (%)	1,230 (37.14)	1,007 (35.57)	223 (46.98)	χ^2^ = 9.90	0.002
Arthritis, yes, *n* (%)	584 (16.31)	377 (12.13)	207 (42.50)	χ^2^ = 100.78	<0.001
Depression, yes, *n* (%)	477 (15.22)	303 (12.02)	174 (35.24)	χ^2^ = 78.23	<0.001
Sleep disorder, yes, *n* (%)	330 (11.41)	210 (9.51)	120 (23.30)	χ^2^ = 71.47	<0.001
Hypertension, yes, *n* (%)	1,060 (29.33)	836 (27.12)	224 (43.14)	χ^2^ = 13.28	<0.001
Diabetes, yes, *n* (%)	449 (10.43)	351 (9.80)	98 (14.40)	χ^2^ = 5.20	0.023
Dyslipidemia, yes, *n* (%)	2,116 (65.84)	1764 (64.64)	352 (73.34)	χ^2^ = 3.78	0.052
CVD, yes, *n* (%)	368 (10.79)	266 (9.59)	102 (18.34)	χ^2^ = 21.10	<0.001
CKD, yes, *n* (%)	270 (6.63)	224 (6.59)	46 (6.83)	χ^2^ = 0.04	0.836
Total energy, kcal, Mean (S.E)	2157.41 (26.03)	2157.89 (26.03)	2154.42 (68.27)	*t* = 0.05	0.960
C-reactive protein, mg/dL, Mean (S.E)	0.34 (0.01)	0.33 (0.01)	0.40 (0.03)	*t* = −2.26	0.038
Glucocorticoids, yes, *n* (%)	46 (1.03)	28 (0.82)	18 (2.32)	χ^2^ = 11.11	<0.001
Analgesics, yes, *n* (%)	279 (7.72)	160 (5.44)	119 (21.98)	χ^2^ = 63.57	<0.001
Osteoporosis, *n* (%)				χ^2^ = 5.28	0.072
No	2,208 (70.97)	1923 (72.00)	285 (64.49)		
Yes	61 (1.29)	56 (1.30)	5 (1.21)		
Unknown	867 (27.74)	697 (26.70)	170 (34.30)		

CLBP, chronic low back pain; PIR, poverty income ratio; MET, metabolic equivalent of task; BMI, body mass index; CVD, cardiovascular disease; CKD, chronic kidney disease.

### Associations between flavonoids intake and CLBP

Table 2 presents the results of logistic regression models examining the associations between total flavonoids and CLBP. Compared with the lowest total flavonoids intake tertile (reference group), participants in highest tertile (>170 mg) were more likely to have a lower likelihood of CLBP status (Model 1: OR = 0.72, 95%CI: 0.59–0.88, *p* = 0.003). After adjusting age, gender, and race, we found that total flavonoids intake with highest tertile was associated with a reduced odds of CLBP (Model 2: OR = 0.66, 95%CI: 0.54–0.82, *p* < 0.001). After further adjusting all covariates, the relationship of total flavonoids intake with CLBP remained statistically significant (Model 3: OR = 0.74, 95%CI: 0.57–0.95, *p* = 0.023). As shown in Supplementary Table S3, we also analyzed the associations of each subtype of flavonoids intake with CLBP. After adjusting all covariates, there was a significant negative association between highest tertile of flavonols and CLBP (Model 3: OR = 0.68, 95%CI: 0.47–0.98, *p* = 0.041). However, no significant associations were observed between isoflavones, anthocyanidins, flavanones, flavones, and flavan-3-ols with CLBP (*p* > 0.05). Subsequently, we further explored the relationship between four specific subtypes of flavonols and CLBP. Notably, when using the first tertile as the reference, the third tertile of kaempferol (Model 3: OR = 0.63, 95%CI: 0.43–0.92, *p* = 0.019) and myricetin (Model 3: OR = 0.62, 95%CI: 0.39–0.98, *p* = 0.042) were associated with a reduced possibility of CLBP in the fully adjusting model.

**Table 2:** Associations between flavonoid intake and CLBP.

Variables	Model 1		Model 2		Model 3	
	OR (95% CI)	*p*	OR (95% CI)	*p*	OR (95% CI)	*p*
Total flavonoids intake						
≤38 mg (the first tertile)	Ref		Ref		Ref	
38 ~ 170 mg (the second tertile)	0.96 (0.72–1.26)	0.734	0.93 (0.70–1.23)	0.593	1.09 (0.76–1.57)	0.612
>170 mg (the third tertile)	0.72 (0.59–0.88)	0.003	0.66 (0.54–0.82)	<0.001	0.74 (0.57–0.95)	0.023

CLBP, chronic low back pain; OR, odds ratio; CI, confidence interval.

Model 1: adjusted for no covariates.

Model 2: adjusted for age, gender, and race.

Model 3: adjusted for age, gender, race, poverty income ratio, arthritis, depression, sleep disorder, and analgesics.

### Stratified analysis

As shown in Table 3, we explored the correlation between flavonoids intake and CLBP across different population stratified by age, sedentary behavior time, arthritis, depression, and sleep disorder. The result suggested that compared to the first tertile, the third quantile of flavonoids intake was significantly related to a decreased likelihood of CLBP among participants aged ≥45 years (OR = 0.52, 95%CI: 0.35–0.76, *p* = 0.002), with sedentary behavior time of >3 h (OR = 0.60, 95%CI: 0.41–0.86, *p* = 0.009), with arthritis (OR = 0.51, 95%CI: 0.29–0.90, *p* = 0.023), depression (OR = 0.48, 95%CI: 0.24–0.98, *p* = 0.043), and sleep disorder (OR = 0.27, 95%CI: 0.12–0.60, *p* = 0.003).

**Table 3:** Stratified analysis based on age, sedentary behavior time, arthritis, depression, and sleep disorder.

Subgroup	OR (95%CI)	*p*	OR (95%CI)	*p*
**Subgroup I: Age**	**Age < 45 (*n* = 1,640)**		**Age ≥ 45 (*n* = 1,496)**	
Flavonoids intake				
≤38 mg (the first tertile)	Ref		Ref	
38 ~ 170 mg (the second tertile)	1.33 (0.73–2.45)	0.332	0.90 (0.48–1.70)	0.734
>170 mg (the third tertile)	1.11 (0.74–1.66)	0.609	0.52 (0.35–0.76)	0.002
**Subgroup II: Sedentary behavior time**	**≤ 3 h (*n* = 1,132)**		**>3 h (*n* = 2004)**	
Flavonoids intake				
≤38 mg (the first tertile)	Ref		Ref	
38 ~ 170 mg (the second tertile)	1.07 (0.52–2.24)	0.839	1.10 (0.70–1.73)	0.651
>170 mg (the third tertile)	1.19 (0.48–2.96)	0.692	0.60 (0.41–0.86)	0.009
**Subgroup III: Arthritis**	**No (*n* = 2,552)**		**Yes (*n* = 584)**	
Flavonoids intake				
≤38 mg (the first tertile)	Ref		Ref	
38 ~ 170 mg (the second tertile)	1.32 (0.74–2.35)	0.329	0.81 (0.39–1.71)	0.566
>170 mg (the third tertile)	0.89 (0.65–1.22)	0.450	0.51 (0.29–0.90)	0.023
**Subgroup IV: Depression**	**No (*n* = 2,659)**		**Yes (*n* = 477)**	
Flavonoids intake				
≤38 mg (the first tertile)	Ref		Ref	
38 ~ 170 mg (the second tertile)	1.24 (0.84–1.85)	0.258	0.88 (0.43–1.76)	0.693
>170 mg (the third tertile)	0.89 (0.66–1.20)	0.413	0.48 (0.24–0.98)	0.043
**Subgroup V: Sleep disorder**	**No (*n* = 2,806)**		**Yes (*n* = 330)**	
Flavonoids intake				
≤38 mg (the first tertile)	Ref		Ref	
38 ~ 170 mg (the second tertile)	1.36 (0.85–2.17)	0.180	0.43 (0.21–0.89)	0.026
>170 mg (the third tertile)	0.95 (0.68–1.33)	0.761	0.27 (0.12–0.60)	0.003

OR, odds ratio; CI, confidence interval.

Adjusted for age (not adjusted in subgroup I), gender, race, poverty income ratio, arthritis (not adjusted in subgroup III), depression (not adjusted in subgroup IV), sleep disorder (not adjusted in subgroup V), and analgesics.

## Discussion

The present study first assessed the association between flavonoids intake and the CLBP risk in the US population, utilizing data from NHANES 2009–2010. The findings indicated the modest inverse associations between total flavonoids intake and likelihood of CLBP. Dietary flavonoids intake was in the highest tertile group, the incidence of CLBP may be lowered by 26% than those in the lowest tertiles after adjusting all covariates. Moreover, this negative correlation remains robust across subgroups aged ≥45 years, with sedentary behavior time > 3 h, with arthritis, depression, and sleep disorder.

CLBP is one of the most common health issues. Notably, dietary patterns have been found to exert a significant impact on the occurrence of CLBP (21). Adherence to the Mediterranean and plant-based diet may effectively reduce musculoskeletal pain (22); The consumption of an unhealthy diet, including higher intake of refined grains, red meat, processed meat, high saturated fat, trans-fatty acids, sweet sugary foods, and caffeine, was associated with an elevated level of inflammatory markers, thereby increasing the risk of CLBP (23). Flavonoids have attracted considerable attention due to their anti-inflammatory and antioxidant properties. These compounds are commonly found in plants, fruits, and vegetables such as cranberries, green tea, black tea, citrus fruits, and grape seeds (24). Several epidemiologic studies have suggested that flavonoid intakes could be beneficial for the prognosis of some diseases and health benefits. For example, Liu et al. reported that higher consumption of dietary flavonoids was associated with periodontal health in the US population (25). A meta-analysis also suggested that a high intake of flavonols, such as quercetin, may potentially mitigate the risk of colon cancer, while a high intake of flavones, such as apigenin, may potentially low the risk of rectal cancer (26). In addition, higher flavonoid intakes may help maintain cognitive function (27). Although studies have also provided some evidence to support the beneficial role of flavonoids in intervertebral disc degeneration (9, 28), but the relationship between dietary flavonoid intake and the CLBP risk remained inconclusive.

In the present study, we adopted the weighted multivariate logistic regression model was to determine the relationship between flavonoids intake and CLBP in a representative population, and found higher total dietary flavonoids intake was associated with decreased CLBP risk after adjusting confounders. The mechanisms underlying the association between dietary flavonoid intake and the CLBP risk are not fully understood. Flavonoids, exhibiting anti-inflammatory and antioxidant properties, stimulate the proliferation of nucleus pulposus cells, downregulate the expression of tumor necrosis factor-*β* and matrix metalloproteinase-3 genes, and enhance the production of bone morphogenetic protein-2, aggrecan, and type II collagen in nucleus pulposus cells. These findings underscore their immense potential in regulating intervertebral disc degeneration and promoting disc regeneration (29, 30). Additionally, in the present study, only flavonols intake exhibited an inverse association with CLBP among all the flavonoid subclasses. While an association between flavonoids and CLBP was observed in this study, it is important to note that not all subtypes of flavonoid compounds have a significant impact on CLBP. Additional clinical studies are needed to verify our findings.

To the best of our knowledge, this is the first study employing a nationally representative sample to elucidate the association between dietary total flavonoids intake and different subclasses of flavonoids with CLBP in U.S. adults, which provided a valuable reference for the prevention strategy of CLBP. However, this study has several limitations. First, this study is a cross-sectional investigation that does not establish a causal relationship between dietary flavonoids intake and CLBP. Second, the NHANES database lacks data on pain severity and different causes of pain, thereby this study cannot further assess the relationship between dietary intake of flavonoids and the severity of CLBP. Third, the participants involved in this study consisted of American adults, potentially influencing the generalizability of the study findings. Finally, the estimation of daily flavonoid intake was based on the average dietary data within 2 days, which may have introduced limitations to the accuracy and representativeness of the results. Further large, prospective studies and clinical trials are now warranted to verify our findings.

## Conclusion

In summary, a higher flavonoid intake was found to be negatively associated with the likelihood of CLBP. For the general adult population, consuming foods rich in flavonoids may be linked to a reduced risk of CLBP, which could be useful in developing strategies aimed at preventing this condition.

## Data Availability

Publicly available datasets were analyzed in this study. This data can be found here: NHANES database, https://wwwn.cdc.gov/nchs/nhanes/.

## Footnotes

^1^ https://wwwn.cdc.gov/Nchs/Nhanes/2009-2010/ARQ_F.htm
