# Supplementary material for: Association between dietary intake of flavonoids and chronic low back pain: a cross-sectional study
Source: Front Nutr. 2024 Oct 3;11:1436461. doi: 10.3389/fnut.2024.1436461 (PMC11484401; doi:10.3389/fnut.2024.1436461)
Supplement: Supplementary file 1 [file Table_1.DOCX]

Supplementary Table 1 Sensitivity analysis on the data before and after interpolation

| Variables | Before interpolation | After interpolation | Statistics | *P* |
| --- | --- | --- | --- | --- |
| Education, n (%) |  |  | χ^2^=2.23 | 0.328 |
| Below high school | 836 (17.33) | 839 (17.33) |  |  |
| High school | 703 (22.58) | 703 (22.54) |  |  |
| University graduate and above | 1591 (60.09) | 1594 (60.14) |  |  |
| Marital status, n (%) |  |  | χ^2^=3.07 | 0.215 |
| Married | 1611 (54.36) | 1613 (54.38) |  |  |
| Never married | 681 (23.34) | 681 (23.33) |  |  |
| Others | 842 (22.30) | 842 (22.29) |  |  |
| PIR, n (%) |  |  | χ^2^=2.60 | 0.273 |
| ≤1.3 | 967 (23.07) | 1083 (23.39) |  |  |
| 1.3~3.5 | 1031 (35.38) | 1143 (35.45) |  |  |
| >3.5 | 846 (41.55) | 910 (41.15) |  |  |
| Drinking, n (%) |  |  | χ^2^=0.14 | 0.706 |
| No | 708 (20.56) | 772 (20.68) |  |  |
| Yes | 2160 (79.44) | 2364 (79.32) |  |  |
| Sedentary behavior time, h, Mean (S.E) | 5.66 (0.11) | 5.66 (0.11) | t=-0.66 | 0.521 |
| BMI, kg/m^2^, Mean (S.E) | 28.78 (0.19) | 28.80 (0.19) | t=-1.7 | 0.101 |
| Arthritis, n (%) |  |  | χ^2^=0.99 | 0.319 |
| No | 2546 (83.74) | 2552 (83.69) |  |  |
| Yes | 581 (16.26) | 584 (16.31) |  |  |
| Depression, n (%) |  |  | χ^2^=2.05 | 0.152 |
| No | 2422 (84.30) | 2659 (84.78) |  |  |
| Yes | 453 (15.70) | 477 (15.22) |  |  |
| CKD, n (%) |  |  | χ^2^=2.67 | 0.102 |
| No | 2743 (93.26) | 2866 (93.37) |  |  |
| Yes | 258 (6.74) | 270 (6.63) |  |  |
| C-reactive protein, mg/dL, Mean (S.E) | 0.34 (0.01) | 0.34 (0.01) | t=-1.23 | 0.238 |

PIR=poverty income ratio; BMI=body mass index; CKD= chronic kidney disease.

Supplementary Table 2 Screening of covariates by weighted univariate logistic regression analysis

| Variables | OR (95%CI) | *P* |
| --- | --- | --- |
| Age |  |  |
| <45 | Ref |  |
| ≥45 | 1.58 (1.09-2.30) | 0.020 |
| Gender |  |  |
| Male | Ref |  |
| Female | 1.04 (0.80-1.35) | 0.773 |
| Race |  |  |
| Non-Hispanic White | Ref |  |
| Non-Hispanic Black | 0.84 (0.56-1.26) | 0.370 |
| Others | 0.64 (0.46-0.90) | 0.014 |
| Education |  |  |
| Below high school | Ref |  |
| High school | 1.21 (0.88-1.67) | 0.219 |
| University graduate and above | 0.68 (0.45-1.04) | 0.071 |
| Marital status |  |  |
| Married | Ref |  |
| Never married | 1.01 (0.74-1.39) | 0.933 |
| Others | 1.65 (1.13-2.39) | 0.012 |
| PIR |  |  |
| ≤1.3 | Ref |  |
| 1.3~3.5 | 0.68 (0.49-0.95) | 0.028 |
| >3.5 | 0.51 (0.32-0.81) | 0.007 |
| Smoking |  |  |
| No | Ref |  |
| Yes | 1.83 (1.30-2.57) | 0.002 |
| Drinking |  |  |
| No | Ref |  |
| Yes | 1.11 (0.81-1.50) | 0.501 |
| Physical activity |  |  |
| <450 MET· min/week | Ref |  |
| ≥450 MET· min/week | 0.93 (0.51-1.66) | 0.785 |
| Unknown | 1.55 (0.82-2.93) | 0.163 |
| Sedentary behavior time |  |  |
| ≤3hours | Ref |  |
| >3hours | 1.25 (0.92-1.69) | 0.142 |
| Obesity |  |  |
| No | Ref |  |
| Yes | 1.60 (1.19-2.17) | 0.004 |
| Arthritis |  |  |
| No | Ref |  |
| Yes | 5.36 (3.90-7.35) | <0.001 |
| Depression |  |  |
| No | Ref |  |
| Yes | 3.98 (2.84-5.59) | <0.001 |
| Sleep disorder |  |  |
| No | Ref |  |
| Yes | 2.89 (2.21-3.78) | <0.001 |
| Hypertension |  |  |
| No | Ref |  |
| Yes | 2.04 (1.36-3.06) | 0.002 |
| Diabetes |  |  |
| No | Ref |  |
| Yes | 1.55 (1.03-2.34) | 0.039 |
| Dyslipidemia |  |  |
| No | Ref |  |
| Yes | 1.50 (0.96-2.36) | 0.072 |
| CVD |  |  |
| No | Ref |  |
| Yes | 2.12 (1.53-2.94) | <0.001 |
| CKD |  |  |
| No | Ref |  |
| Yes | 1.04 (0.70-1.54) | 0.837 |
| Total energy | 1.00 (1.00-1.00) | 0.960 |
| C-reactive protein | 1.18 (1.01-1.38) | 0.043 |
| Glucocorticoids |  |  |
| No | Ref |  |
| Yes | 2.87 (1.43-5.74) | 0.005 |
| Analgesics |  |  |
| No | Ref |  |
| Yes | 4.90 (3.22-7.45) | <0.001 |
| Osteoporosis |  |  |
| No | Ref |  |
| Yes | 1.04 (0.20-5.44) | 0.960 |
| Unknown | 1.43 (1.11-1.85) | 0.008 |

PIR=poverty income ratio; MET=metabolic equivalent of task; BMI=body mass index; CVD=cardiovascular disease; CKD= chronic kidney disease.

Supplementary Table 3 Associations between subtypes of flavonoids intake and CLBP

| Variables | OR (95%CI) | *P* |
| --- | --- | --- |
| **Subtypes of flavonoids** |  |  |
| Isoflavones, mg |  |  |
| 0 | Ref |  |
| ≤ 0.048 | 0.96 (0.64-1.45) | 0.847 |
| > 0.048 | 1.23 (0.92-1.64) | 0.147 |
| Anthocyanidins, mg |  |  |
| ≤0.55 | Ref |  |
| 0.55~8.25 | 1.53 (1.00-2.36) | 0.052 |
| > 8.25 | 1.24 (0.86-1.78) | 0.240 |
| Flavanones, mg |  |  |
| ≤ 0.20 | Ref |  |
| 0.20~7.78 | 1.05 (0.64-1.73) | 0.835 |
| > 7.78 | 1.13 (0.65-1.99) | 0.645 |
| Flavonols, mg |  |  |
| ≤ 9.66 | Ref |  |
| 9.66~20.41 | 0.90 (0.63-1.29) | 0.545 |
| > 20.41 | 0.68 (0.47-0.98) | 0.041 |
| Flavones, mg |  |  |
| ≤ 0.33 | Ref |  |
| 0.33~0.93 | 0.90 (0.56-1.44) | 0.641 |
| > 0.93 | 0.80 (0.51-1.27) | 0.322 |
| Flavan-3-ols, mg |  |  |
| ≤ 8.33 | Ref |  |
| 8.33~69.47 | 1.35 (0.96-1.90) | 0.084 |
| > 69.47 | 0.88 (0.69-1.12) | 0.276 |
| **Subtypes of flavonols** | | |
| Isorhamnetin, mg |  |  |
| ≤ 0.27 | Ref |  |
| 0.27~0.91 | 0.79 (0.57-1.09) | 0.140 |
| > 0.91 | 0.92 (0.54-1.57) | 0.750 |
| Kaempferol, mg |  |  |
| ≤1.61 | Ref |  |
| 1.61~4.76 | 0.91 (0.68-1.21) | 0.487 |
| > 4.76 | 0.63 (0.43-0.92) | 0.019 |
| Myricetin, mg |  |  |
| ≤ 0.38 | Ref |  |
| 0.38~1.31 | 1.12 (0.69-1.81) | 0.622 |
| > 1.31 | 0.62 (0.39-0.98) | 0.042 |
| Quercetin, mg |  |  |
| ≤6.16 | Ref |  |
| 6.16~13.14 | 0.86 (0.66-1.12) | 0.234 |
| > 13.14 | 0.78 (0.53-1.16) | 0.210 |

CLBP=chronic low back pain; OR=odds ratio; CI=confidence interval.

Adjusted for age, gender, race, poverty income ratio, arthritis, depression, sleep disorder, and analgesics.
